# Head and neck exam practices of dental professionals

**DOI:** 10.1002/cre2.772

**Published:** 2023-09-27

**Authors:** Sarah Glass, Vanessa Brown, Caroline Carrico, Parthasarathy Madurantakam

**Affiliations:** ^1^ Oral Diagnostic Sciences VCU School of Dentistry Richmond Virginia USA; ^2^ Dental Student VCU School of Dentistry Richmond Virginia USA; ^3^ Dental Public Health and Policy VCU School of Dentistry Richmond Virginia USA; ^4^ General Practice VCU School of Dentistry Richmond Virginia USA

**Keywords:** dentists, oral cancer screening, practice patterns

## Abstract

**Objective:**

Periodic examination of the head and neck includes screening for oral cancer, which is largely performed in dental offices by vigilant oral healthcare providers. The aim of this study was to assess practice patterns among Virginia dentists in performing head and neck exams and the referral rates of biopsies after completion of head and neck exams. We hypothesized that not all dentists perform head and neck exams and there is a difference between dentists who refer patients for a biopsy and those that perform biopsies.

**Methods:**

General dentists and dental specialists who are members of the Virginia Dental Association were invited to participate in a cross‐sectional survey study through REDCap to self‐report their head and neck exam protocols.

**Results:**

A total of 224 providers completed the survey. The majority of respondents were general dentists with more than 20 years in practice, who practice in a private setting, and see more than 10 patients in a day. All respondents stated they perform intraoral examinations, but 10 respondents stated they do not perform extraoral examinations. Nearly a third of respondents reported doing their own biopsies.

**Conclusions:**

Although only 8.5% of oral healthcare providers in Virginia responded to our survey, respondents are following the 2017 ADA good practice statement by providing their patients with head and neck exams to screen for oral cancer. Additional education pertaining to extraoral anatomy, malignant transformation of oral potentially malignant disorders, and pathology procedures may be helpful to clinicians.

## 1 INTRODUCTION

The incidence of oral and oropharyngeal cancer is rising, and the American Cancer Society estimates 54,540 new cases will be diagnosed in the United States in 2023 (Key Statistics for Oral Cavity and Oropharyngeal Cancers). The 5‐year survival depends on the stage at the time of diagnosis with a later‐stage diagnosis harboring a decreased survival rate. Approximately 70% of all new cases are diagnosed at a later stage (Lingen et al., 2017).

The oral cavity lends itself well to cancer screening with a visual and tactile examination. Examinations are minimally invasive, do not require special equipment, and can be completed in a short period of time. Despite these advantages, shortcomings have been identified such as variation in examiner knowledge/training/calibration and lack of understanding related to the malignant transformation of lesions (Speight et al., 2017). Currently, there are no actionable recommendations for population‐based oral cancer screenings from policymakers and public stakeholders. For asymptomatic adults aged 18 years or older, the US Preventative Task Force states there is insufficient evidence to recommend screening for oral cancer by primary care providers (Moyer & US Preventive Services Task Force, 2014). Oral cancer screenings are largely performed as part of good clinical practice in dental offices by vigilant oral healthcare providers (Gupta et al., 2019; Walsh et al., 2021; Warnakulasuriya & Kerr, 2021). In 2017, the American Dental Association (ADA) supported the following recommendation: “Clinicians should obtain an updated medical, social, and dental history and perform an intraoral and extraoral conventional visual and tactile examination in all patients.” (Lingen et al., 2017) The National Center for Health Statistics reports that 62.7% of adults aged 18 and over had a dental visit in 2019 (Cha & Cohen, 2022). Of this population visiting the dentist, it is unclear what percentage is receiving a head and neck exam.

A head and neck exam consists of an extraoral and intraoral component. In 2019, the ADA expanded the policy on oral cancer detection to include evaluation for oropharyngeal cancer (American Dental Association, 2019). Evaluation of head and neck lymph nodes is a critical component of the extraoral exam as painless cervical lymphadenopathy is the most common clinical presentation for patients with human papillomavirus‐associated oropharyngeal squamous cell carcinoma (McIlwain et al., 2014). In 2020, Orofacial Pain and Oral Medicine, disciplines with emphasis on head and neck exams, became recognized by the American Dental Association as dental specialties. The Academy of Orofacial Pain recently released dental school curriculum recommendations to include more emphasis on the evaluation of muscles and temporomandibular joints (Chen et al., 2021).

Oral potentially malignant disorders (OPMD) are defined as “any oral mucosal abnormality that is associated with a statistically increased risk of developing oral cancer” (Warnakulasuriya et al., 2021). When detected clinically on an intraoral exam, an immediate biopsy or referral to a specialist is recommended, and a definitive diagnosis is achieved by a tissue biopsy (Lingen et al., 2017). Clinical decision‐making for suspicious oral lesions can be challenging for dental providers. Kerr et al. (2020) found fewer than 40% of dentists have a decision‐making policy for suspicious oral lesions. Gaballah, Kamis, et al. found that the overall accuracy of diagnosis for dental students and recent graduates evaluating oral lesions clinically was 46% (Gaballah et al., 2021). Furthermore, the natural history of each OPMD is not completely understood leading to continuous research and re‐evaluation. An example of an OPMD that can be challenging to recognize is proliferative verrucous leukoplakia (PVL). A study by Tarakji showed that general dentists did not recognize PVL and its higher malignant transformation potential as readily as dental specialists (Tarakji, 2022).

The aim of this study was to assess practice patterns among Virginia dentists in performing head and neck exams and the referral rates of biopsies after completion of head and neck exams. We hypothesized that not all dentists perform head and neck exams and there is a difference between dentists who refer patients for a biopsy and those that perform biopsies. Insights gained from this study would serve to identify opportunities to close gaps in the implementation of the 2017 ADA‐supported good practice statement.

## 2 METHODS

Virginia Commonwealth University IRB reviewed and determined the research study qualified for exemption (HM20024050). The Massey Cancer Center Protocol Review and Monitoring Committee approved the research study by expedited review (MCC‐21‐19123). Participants were informed of the purpose of the study, that their responses were anonymous, participation was voluntary, and that it would take approximately 5–7 min. None of the questions were mandatory and responses were only stored if the survey was submitted.

The design of this project was a cross‐sectional survey study of currently practicing dentists who are members of the Virginia Dental Association (VDA) in any field of specialty. Dental hygienists were excluded. The survey was administered using Research Electronic Data Capture (REDCap). REDCap is a secure, web‐based software platform designed to support data capture for research studies. The survey was an original, 17‐item questionnaire developed by a dental student, general dentist, and oral pathologist to gather preliminary information on practices related to head and neck exams. It did not intend to cover all aspects of practice. Before dissemination, the survey was tested internally for clarity, completeness, and approximate time required. All questions were categorical and varied from being nominal, ordinal, and binary in nature. The survey began by obtaining participant demographics including the number of years they have been in practice, the field of dentistry they are currently practicing in, the volume of patients they see on a normal workday, and the setting they currently practice in. Later questions inquired about the participant's protocol when it comes to performing head and neck exams as well as handling biopsies. Participants were asked specifically about extraoral exams, intraoral exams, office protocol on exam frequency, biopsy practices, referral practices, and frequency of malignant biopsy results. Branching logic was used, so questions only appeared if they were relevant to the respondent.

The newsletter containing the invite and link to the survey was initially emailed on March 29, 2022 and a reminder email was sent on May 19, 2022. The first was sent to 3321 addresses and had a 63% open rate (*n* = 2092) and the second was sent to 3401 with a 51% open rate (*n* = 1735). For the sake of response rate, we considered the denominator to be 2613 based on an estimated 70% overlap among those who opened both newsletters (2092 + (0.3 × 1735) = 2613). In an attempt to improve the response rate, the survey was mentioned in a podcast regarding healthcare and advertised to local dental study clubs. Additionally, it was purposely presented during the month of April due to the momentum around Oral Cancer Awareness Month. The survey was closed on June 13, 2022.

Responses were summarized using counts and percentages to quantify practice patterns across Virginia. Associations between self‐reported practices (performing head and neck exams, taking biopsies) and provider characteristics (provider type, years in practice, approximate patients per day) were assessed using chi‐squared tests. The significance level was set at 0.05. SAS EG v.8.2 (SAS Institute) was used for all analyses.

## 3 RESULTS

### 3.1 Provider practice information

A total of 224 providers completed the survey (approximately an 8.5% response rate). The majority of respondents were general dentists (*n* = 173, 77%) with more than 20 years in practice (*n* = 125, 56%), practice in a private setting (*n* = 203, 91%), and see more than 10 patients in a day (*n* = 180, 81%). The complete demographics of respondents are provided in Table 1.

**Table 1:** Respondent characteristics.

	*n*	%
Years in practice		
Less than 5 years	26	12
5–10 years	30	13
10–15 years	21	9
15–20 years	21	9
More than 20 years	125	56
Speciality		
General dentistry	173	77
Other	51	23
Patients per day		
Less than 5	4	2
5–10	39	17
More than 10	180	81
Practice setting^a^		
Private practice	203	91
Hospital	13	6
Academia	13	6
Public health	10	4
Other	7	3

^a^ Respondents could select all that apply.

### 3.2 Head and neck exam practices

All respondents reported examining intraoral sites, and 96% (*n* = 213) reported examining extraoral sites. Most respondents indicated that their office protocol is to perform a head and neck exam every 6 months (*n* = 137, 61%) or at every visit (*n* = 45, 20%). Interestingly, three respondents (1%) indicated that their office protocol is never for head and neck exam frequency, but they self‐reported performing exams. A summary of respondent head and neck exam practices is listed in Table 2.

**Table 2:** Head and neck exam practices.

	*n*	%
Office protocol for frequency		
At every visit	45	20
Every 3 months	4	2
Every 6 months	136	61
Once a year	17	8
Never	3	1
Other	19	8
Examine intraoral sites		
Yes	224	100
No	0	0
Examine extraoral sites^a^		
Yes	213	96
No	10	4

^a^ One respondent did not answer regarding extraoral sites.

### 3.3 Biopsy practices

**Figure 1:**
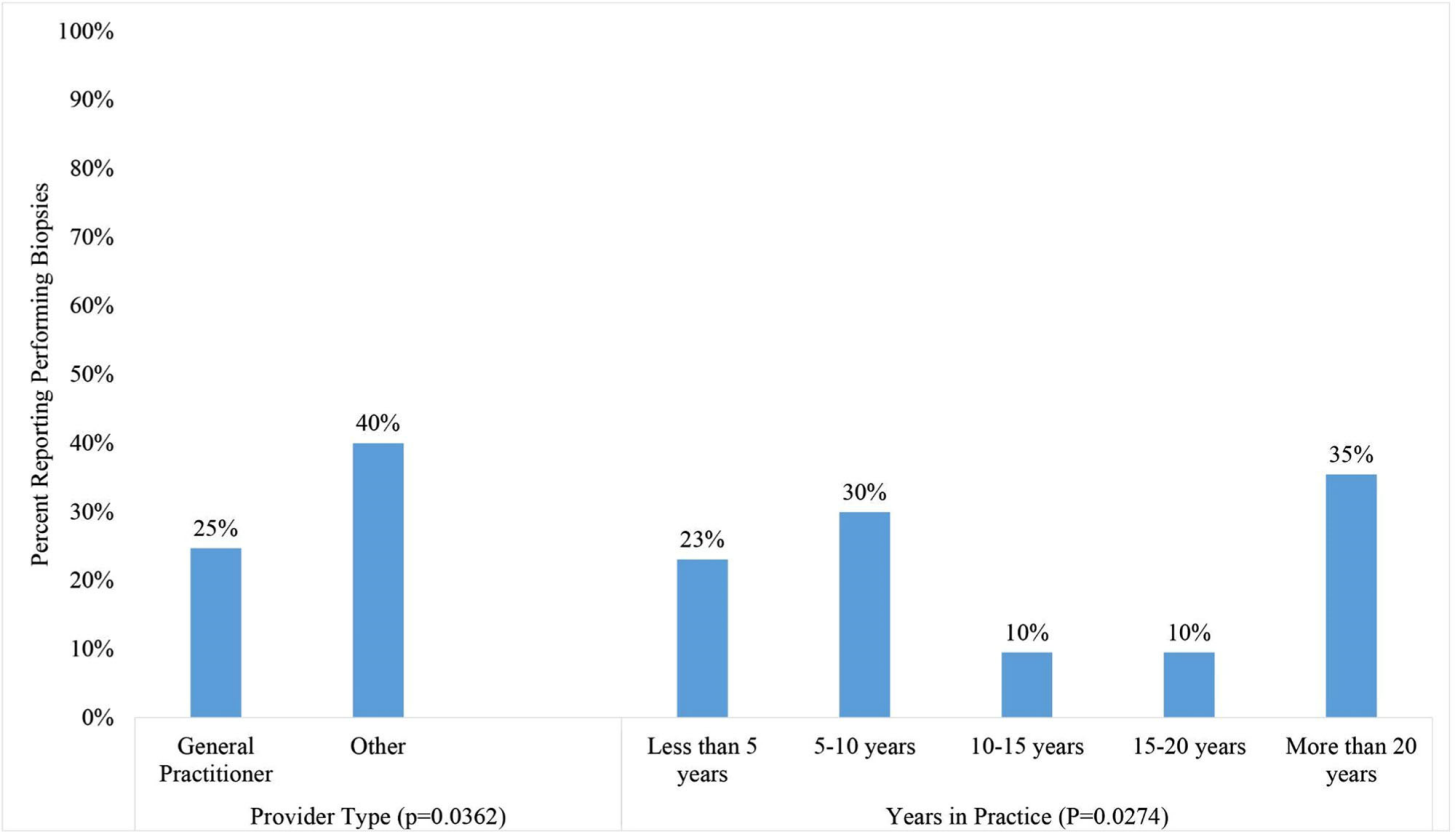
Performing biopsies by provider type and years in practice.

Nearly a third of respondents reported doing their own biopsies (*n* = 64, 29%). Eighty‐nine percent reported conducting less than 5 per month (*n* = 57) (Table 3). There were significant associations between years in practice (*p* = .0274) and provider type (*p* = .0362) (Figure 1). Approximately 10% of respondents with 10–15 or 15–20 years in practice reported performing biopsies compared to 23–35% of those with less than 5 years, 5–10, or more than 20 years in experience. Among general practitioners, 25% self‐reported performing their own biopsies compared to 40% of other providers. Ninety‐seven percent reported that biopsies rarely (75%) or never (22%) come back as malignant (Table 3).

**Table 3:** Perceived biopsy trends.

	*n*	%
Approximately how many times in the past 5 years have you referred a patient for a biopsy?		
0	11	5
1–3	48	22
3–6	41	18
6–9	51	23
More than 9	71	32
How often do the biopsies come back as a malignant oral finding?		
Never	46	22
Rarely	160	75
Frequently	6	3
Do you perform your own biopsies on lesions		
No	160	71
Yes	64	29
Approximately how many biopsies do you conduct monthly?		
<5	57	89
6–10	6	9
>10	1	2

### 3.4 Referral practices

**Figure 2:**
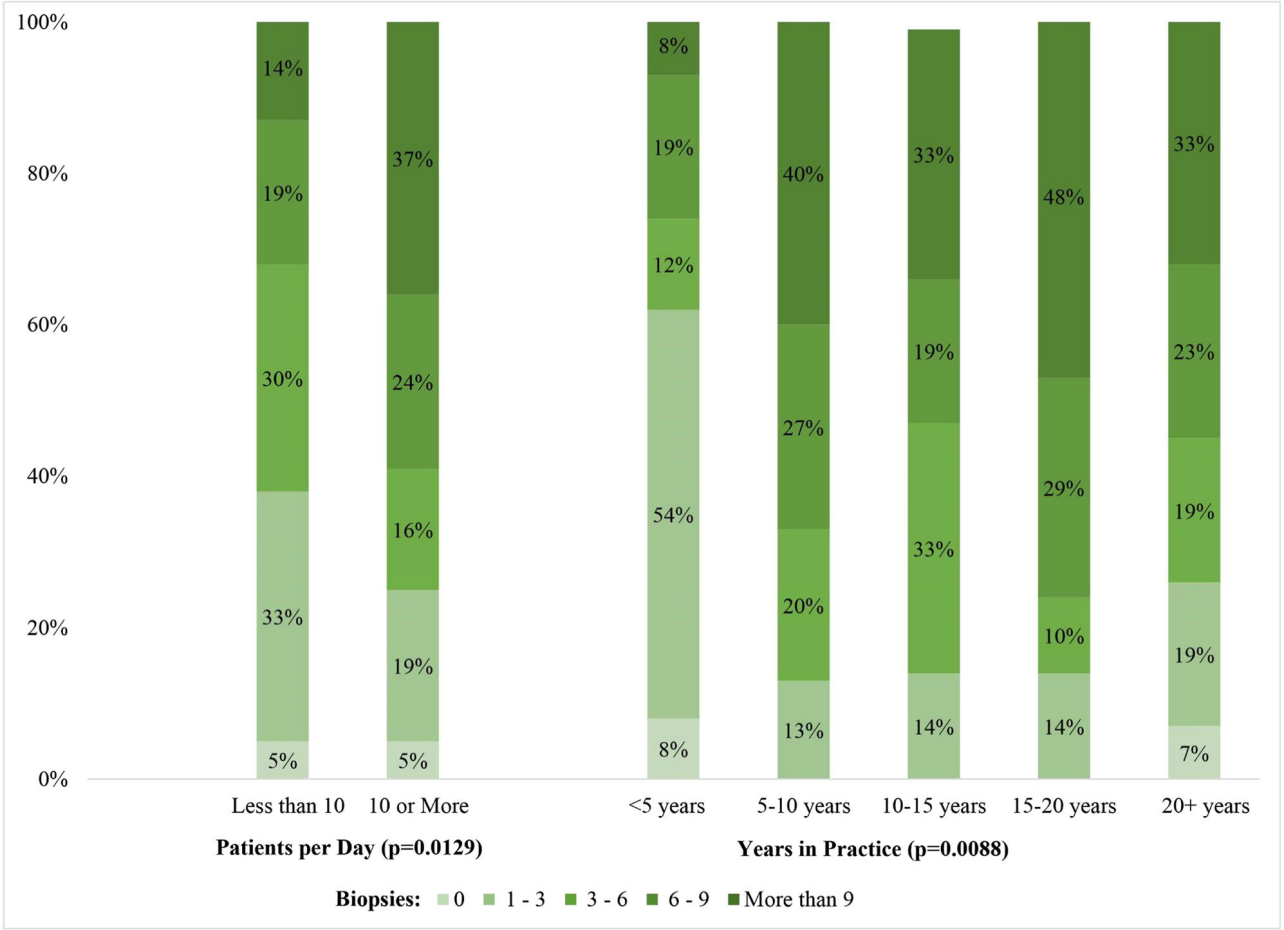
Number of referrals for biopsies by patients per day and years in practice.

The number of referrals providers reported in the past 5 years was significantly associated with the number of patients they see in a typical day (*p* = .0129) and the number of years in practice (*p* = .0088) (Figure 2). Those who reported more patients per day tended to be more likely to report more referrals. For example, 37% of those who reported seeing more than 10 patients per day reported having referred more than nine for biopsies, compared to 14% of those who see less than 10 patients per day. For those with less than 5 years in practice, only 8% reported having referred more than nine patients for biopsy compared to 33%–48% among those with more than 5 years in practice. Only 5% of respondents (*n* = 11) reported having never referred a patient for a biopsy (Table 3). Of these 11 respondents, five were oral and maxillofacial surgeons who reported performing their own biopsies. Interestingly, the remaining six were general practitioners, an orthodontist, and a prosthodontist who all reported not performing biopsies.

## 4 DISCUSSION

In this study, 100% of surveyed dental respondents report performing intraoral head and neck examinations on their patients. The observed variation in office protocol frequency is expected given no formal recommendations. The results are encouraging but should be interpreted with caution due to potential biases with self‐reporting. A survey study by Golburean et al. demonstrated that most of their survey participants (88.0%, 94.6%, and 72.0%) reported that they examine all new patients for oral mucosal lesions (Golburean et al., 2021). Also an encouraging result, but they describe shared concerns with accuracy. A survey study by Kujan et al. showed that 92% of their respondents reported performing a systemic oral examination in patients older than 40 (Kujan et al., 2006). This percentage decreased slightly for a younger patient population (81.4%); interestingly, they observed a perceived incidence of oral cancer trending upwards in younger female patients (Kujan et al., 2006). Survey respondents provided a lack of training, knowledge, and expertise as a common barrier to performance (Golburean et al., 2021).

Prior patient‐based survey studies conducted in Maryland, North Carolina, and Florida using computer‐assisted telephone surveys revealed that 28%, 29%, and 19.5% of participants, respectively, reported receiving an oral cancer screening exam (Horowitz et al., 1998; Patton et al., 2004; Tomar & Logan, 2005). A study analyzing data from the 2011–2016 National Health and Nutrition Examination Survey (NHANES) based on people 30 years old or older who visited a dentist in the last 2 years, showed that 37.6% of individuals reported receiving an intraoral exam and 31.3% of individuals reported receiving an extraoral exam (Gupta et al., 2019). The vast differences between survey results of dentists and patients regarding the same topic highlight the challenge of accurately characterizing dental practices. If dentists explicitly notify patients that they are performing an oral cancer screening, then this may help illuminate the practice to patients.

Although all respondents stated they perform intraoral examinations, 10 respondents stated they do not perform extraoral examinations. The NHANES Survey also noted this discrepancy (Gupta et al., 2019). This may be due to the lack of knowledge and training pertaining to the assessment of extraoral anatomy.

In addition to asking providers to self‐report their head and neck exam performance, questions related to referrals and biopsies were asked to further elucidate the ability to detect lesions. In this study, most respondents (95%) report referring patients for a biopsy. Providers who see multiple patients a day and who have been in practice for many years reported having sent the most referrals. This may be explained by experienced, busy practitioners, having well‐established referral patterns. New providers may have a limited referral network (Specialty Referrals; American Dental Association, 2023). Other studies looking more specifically at referral patterns from general dentists show that patients with suspicious oral lesions are often referred to oral and maxillofacial surgeons and oral medicine clinicians (Kujan et al., 2006).

Nearly a third of respondents (29%) reported doing their own biopsies. These providers were more likely to be dental specialists with more than 20 years of experience. In a survey by Diamanti et al., both surgeons and dentists suggested that training in biopsy technique, improved pathology support, and improved fees would help facilitate more biopsy procedures in practice (Diamanti et al., 2002). The role of a pathologist in healthcare can be elusive. Studies surveying students suggest that students do not feel exposed to pathology during their education (McCloskey et al., 2020; Saluja et al., 2020). Providing visibility to pathology during education may help highlight interactions between pathologists and clinical practitioners for improved patient care.

Most respondents (78%) reported a biopsy result returning as malignant, albeit rarely for most. Psoter et al. surveyed general dentists who were members of the US National Dental Practice‐Based Research Network and found that during the preceding 6 months, 87% of dentists reported discovering at least one suspicious lesion, and 15% of dentists reported at least one lesion histologically confirmed as oral cancer (Psoter et al., 2019). With the majority of respondents detecting a malignant lesion in their career, continuing education on the topics of OPMDs and oral cancer is beneficial. Additionally, the trends in cancer can change over time further emphasizing the need to stay current with continuing education. Today there is an increase in human papillomavirus‐associated oropharyngeal squamous cell carcinoma and tongue squamous cell carcinoma in young females (Chaturvedi et al., 2011; Ng et al., 2017).

This study is limited due to the small sample size, regional restrictions, and self‐reporting. Future research looking at head and neck exams with a larger sample size would be beneficial as well as investigating the quality of head and neck exams performed. Other studies examining the detection of OPMDs and clinical decision‐making skills could provide clarity to the steps following a head and neck exam.

Although only 8.5% of oral healthcare providers in Virginia responded to our survey, respondents are following the 2017 ADA good practice statement by providing their patients with head and neck exams to screen for oral cancer. Additional education pertaining to extraoral anatomy, malignant transformation of oral potentially malignant disorders, and pathology procedures may be helpful to clinicians.

## AUTHOR CONTRIBUTIONS

**Sarah Glass**: Conceptualization; investigation; original draft writing; supervision. **Vanessa Brown**: Conceptualization; survey creator; investigation; draft editing. **Caroline Carrico**: Statistical analysis; draft editing. **Parthasarathy Madurantakam**: conceptualization; draft editing.

## CONFLICT OF INTEREST STATEMENT

The authors declare no conflict of interest.

## Data Availability

Data are available on request from the authors.
